# The Welfare versus Work Paradox

**DOI:** 10.1371/journal.pone.0321564

**Published:** 2025-05-06

**Authors:** Roberto Iacono

**Affiliations:** 1 Department of Social Work (ISA), Norwegian University of Science and Technology (NTNU), Trondheim, Norway; 2 International Inequalities Institute (III), London School of Economics and Political Science (LSE), London, United Kingdom; 3 CESifo Research Network, CESifo, Munich, Germany; University of Verona: Universita degli Studi di Verona, ITALY

## Abstract

How can countries balance work incentives and access to welfare without violating the principle that work shall always be strictly preferred to welfare? In a context in which wages stagnate or drop, and benefit levels are reduced due to austerity measures, the welfare versus work paradox arises. This research shows analytically that when both wages and benefits approach the subsistence level, welfare becomes preferable to work, violating the work incentive principle. The policy implication of this result is that, to maintain the validity of the work incentive principle, minimum wages must be kept above the subsistence threshold.

## Introduction

The stagnation in real wages is a widespread phenomenon across many advanced economies, with the lowest wages in some countries gradually declining to subsistence levels^1^ (see [1] for Italy; [2] for the UK; [3] for the US). Simultaneously, the generosity of minimum income schemes - meaning their ability to provide the means for a standard of living above subsistence - has been curtailed by austerity measures (see [4] for the US; [5] for the Scandinavian countries; [6] for a comparative overview on minimum income schemes in European economies).

This simultaneous decline in real wages at the bottom of the distribution, and in welfare benefit levels, both converging toward the subsistence income threshold, poses a challenge to the work incentive principle - which stipulates that employment shall always be preferred to welfare [7] - and exposes a formal paradox.

To the best of our knowledge, this paradox has not been illustrated analytically before. Specifically, when both employment and welfare support converge near subsistence, the work incentive principle no longer holds. Under fairly general assumptions about (i) individual preferences, and about (ii) the relative effort required to achieve a subsistence income level, welfare may indeed become the preferable option.

This paradoxical result leads to neat policy implications, that inform the current debate on the way minimum wages should be designed in advanced economies [8]. The result suggests that, for the work incentive principle to remain valid and govern incentives in the right direction, minimum wages must be set and maintained consistently above the subsistence threshold.

Ideally, the gap between minimum wages and the subsistence level should be sufficiently large to accomodate for a positive degree of *welfare generosity* (meaning in-cash minimum income floors intentionally at a higher level than the subsistence level) for countries that choose to allocate resources toward it. This possibility is not contemplated in [9], since in their framework the function of welfare benefits is to bring individuals to a subsistence income floor, but not beyond.

The next methods section illustrates the paradox using a simple analytical framework. The results section presents the findings; whilst the discussion section elaborates on the policy implications, and concludes.

## Methods: Modeling the welfare versus work paradox

This section introduces the model. The model is kept simple and tractable, and it builds on fairly general assumptions. We consider a population of *n* individuals, with a dominant fraction  ( 0 < *k* < 1 )  actively participating in the labor market (the labor force), while the remaining fraction (1–*k*) includes passive welfare recipients (i.e., welfare recipients not participating in any job search programs).

Assume that $w_{i}>0$ is real wage from work for individual *i*, $b_{i}>0$ is the real level of benefit from the specific means-tested minimum income program (often labeled as social assistance) the agent is taking part in, while $s_{i}>0$ indicate the subsistence or minimum income level, all expressed in a common currency and in real terms.

Notice that we allow in principle the benefit level $b_{i}$ for individual *i* to be higher than the subsistence level $s_{i}$, which we term as the degree of *welfare generosity*. The government might therefore decide to hold the benefit level above subsistence, namely $b_{i}\geq s_{i}>0$. This can be coherent with the rationale for minimum income programs, supporting recipients to keep their disposable income above the minimum level of income barely sufficient to survive. A positive degree of welfare generosity does not need to create incentive problems, since the work incentive principle on the other hand ensures that utility from work exceeds utility from receiving benefits ($U_{i}(w)\geq U_{i}(b)$).

Notice that by allowing $b_{i}\geq s_{i}>0$, the model in this paper deviates from the framework in [9], in which the government assists only those who earn less than the minimum or subsistence level of income, in order to reach the latter. We deem the assumption in our model as more realistic, as minimum income programs in advanced economies can indeed allow a level of disposable income that exceeds the subsistence level.

### Preferences over work, leisure and welfare

Let us define preferences for the two type of agents in the model. The fraction *k* of individuals in the labor market have standard quasi-linear preferences over wage income from work *w*, effort *e*(*w*) and leisure *l*, given by:


$$\begin{array}{ccc} U_{k}(w,l,e)=w^{\gamma }+l-e(w). & & \end{array}$$ (1)

*γ* ∈ ( 0 , 1 )  implies diminishing utility from income. *e*(*w*) is effort required for work where *e*(.) is increasing and strictly convex, namely $e^{\prime }(w)>0$ and $e^{\prime \prime }(w)>0$ (i.e., as wages increase, the relative effort increases). Since preferences are non-linear with respect to wage income from work and the effort function *e*(.) is also non-linear, the only linear component is leisure *l*. Notice that this assumption is not decisive for the results of the model. Finally, in this standard quasi-linear preference model, the utility derived from work and leisure is structured such that the opportunity cost of leisure is explicitly represented. This formulation ensures that the marginal value of leisure is directly linked to the wage rate while remaining independent of wealth effects.

The same quasi-linear preferences yield for the remaining individuals that are on welfare programs (fraction 1–*k*). Namely, welfare recipients derive utility from in-cash benefit from a welfare program *b* and from leisure *l*^2^, net of the (linear) disutility from the cost of applying for the specific benefit *c*:


$$\begin{array}{ccc} U_{1-k}(b,l,c)=b^{\delta }+l-c. & & \end{array}$$ (2)

*δ* ∈ ( 0 , 1 )  assigns diminishing utility for a higher welfare benefit level. For simplicity, we do not allow in this model individuals to be both workers and benefit recipients or to shift from one group to the other.^3^ The reason is that we wish to pin down the comparison between working and being on welfare, using the same identical and fairly general quasi-linear preferences.

In other words, if an individual finds it optimal to claim the benefit given the circumstances, we disregard the possibility that the same individual also supplies some labor to the market.^4^ On the other hand, if an agent receives wage income from work, we assume that this person is by default not entitled to any benefits from the welfare state. This assumption keeps the illustration of the welfare versus work paradox more neat. A more fully-fledged version of this model could expand to allow individuals to combine the two settings.

### The work incentive principle

The work incentive principle is crucial in this framework, as it maintains incentives to work while balancing the extent of welfare generosity, with the latter increasing (or being reduced) as the gap between benefit levels and the subsistence income $b_{i}\geq s_{i}$ widens (or shrinks).

In brief, the work incentive principle states that work shall be economically more convenient than being on welfare [9]. We can translate this into our framework by stating that the utility from working must be greater than or equal to the utility derived from receiving welfare:


$$\begin{array}{ccc} U_{k}(w,l,e)\geq U_{1-k}(b,l,c). & & \end{array}$$ (3)

Substituting the utility functions from Eqs 1 and 2 into this general formulation gives back:


$$\begin{array}{ccc} w^{\gamma }+l-e(w)\geq b^{\delta }+l-c. & & \end{array}$$ (4)

Simplifying by deleting the leisure term on both sides returns:


$$\begin{array}{ccc} w^{\gamma }-e(w)\geq b^{\delta }-c. & & \end{array}$$ (5)

The next section will present the analytical conditions under which the welfare versus work paradox arises.

## Results: Analytical conditions for the welfare versus work paradox

We have stated until now that $b_{i}\geq s_{i}$ ensures a positive degree of *welfare generosity*, while on the other hand the work incentive principle maintains economic incentives to work since it ensures that $U_{k}(w,l,e)\geq U_{1-k}(b,l,c)$.

Now, maintain that $b_{i}\geq s_{i}$ but imagine that due to a worsening of the labor market conditions, wages drop and start to approach subsistence. Since benefit levels cannot be higher than wages due to the work incentive principle, they will also be pressed downwards, and we will have that both w→s and b→s; hence the condition stated in Eq 5 becomes:


$$\begin{array}{ccc} s^{\gamma }-e(s)\geq s^{\delta }-c. & & \end{array}$$ (6)

For simplicity, we add the assumption that 0 < *γ* = *δ* < 1 so that utility derived from work income and from welfare benefits diminishes at the same speed. Now, Eq 6 leads to the paradox: as both work income and welfare income approach the subsistence level *s*, and assuming realistically that the effort from working an income equal to the subsistence level is higher than the cost *c* of claiming a benefit that earns the same level (*e* ( *s* ) ≥ *c*), then being on welfare becomes more attractive. Specifically, with (0 < *γ* = *δ* < 1) and if and only if (*e* ( *s* ) ≥ *c*), we have that:


$$\begin{array}{ccc} s^{\gamma }-e(s)\leq s^{\delta }-c. & & \end{array}$$ (7)

This implies that welfare provides a higher utility than working, violating the work incentive principle. In other words, under subsistence-level conditions, the disutility from effort (relatively stronger than the disutility from claiming benefit at subsistence levels) can make work less attractive than welfare, violating the intended hierarchy where work should always be preferable.

**Fig 1:**
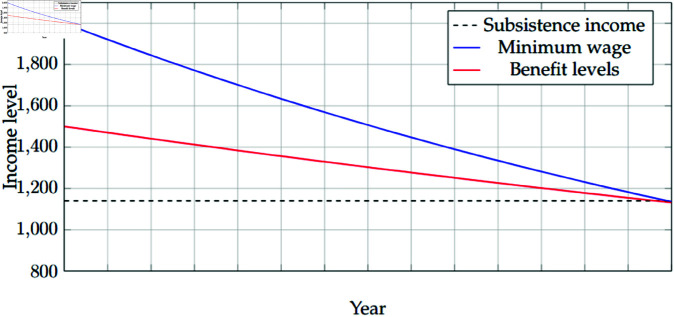
The Welfare versus Work Paradox. This figure illustrates the paradox graphically. The blue line represents the declining real wages, and at any point in time, it guarantees a higher living standard than welfare transfers (red line). This corresponds to the work incentive principle. Both lines are at any point in time above the black line, which indicates the subsistence level (kept constant for simplicity). Benefit levels from transfers are also decreasing due to a decline in welfare generosity. In the final period of time, the red and blue lines become tangent with the black line, leading to the paradox.

The non-linear utility functions for both work and welfare might further exacerbate the problem, especially if we relax the assumption that 0 < *γ* = *δ* < 1 by allowing *γ* < *δ*, meaning that utility from work diminishes at a faster pace than the utility from welfare. A simple graphical illustration of the paradox is offered in Fig 1.

## Discussion and concluding remarks

The model presented in this paper introduces the analytical conditions under which the welfare versus work paradox emerges. We label this condition as the welfare versus work paradox, since it highlights the analytical conditions under which a clear conflict arises between the work incentive principle and welfare. This paradox highlights an inconsistency insofar as the work incentive principle - that work should be economically preferable to welfare - does not deliver a situation that makes the agent better off in terms of utility.

This paradox arises specifically when (i) the lowest wages in the labor market and (ii) the diminishing benefit levels from welfare programs converge around the subsistence income level. Additionally, we assume that the effort required to earn an income equal to subsistence exceeds the cost of claiming a benefit at that same level. Under these conditions, welfare becomes a more attractive option than employment, thereby violating the work incentive principle and activating the paradox.

This suggests the following policy implications for the work incentive principle to remain valid: the wage floor in the labor market (the real minimum wages) must be kept consistently above the subsistence threshold at any point in time. This policy insight should be considered among the other features of the minimum wage design that are currently being considered in this ongoing debate [8,10].

Ideally, the gap between the lowest wages in the labor market (or the minimum wages, when present and institutionally designed) and the subsistence level should be sufficiently large to allow for a positive degree of welfare generosity, namely benefit levels allowing a higher standard of living than the subsistence level.

Future research should advance these findings along several lines: (i) enhancing the model by permitting agents to combine work and welfare benefits or transition between the two groups; (ii) introducing heterogeneous effort costs across individuals, allowing for different abilities and varying opportunity costs of time; and (iii) empirically estimating the effects of the identified paradox, aiming to determine whether welfare indeed becomes the preferred option over work in contexts where the lowest wages and benefit levels converge at the subsistence threshold.

## Footnotes

1 Subsistence level here refers to an income level (in real terms, hence controlling for the price level) barely sufficient for individuals to meet their basic needs for food, shelter, and clothing in the country they reside.

2 These agents dedicate their work hours to participation in welfare programs and have the same amount of leisure time as the first group. We have not specified the utility of leisure to be different for the two groups.

3 We are aware that a significant portion of the population in welfare states belongs to a hybrid group with positive earnings *and* receiving welfare benefits. The assumption of focusing of two non-intersecting ideal-types is grounded purely on modeling justifications.

4 Notice that although the individual is only engaged in wage-remunerated activities, they may actively participate in other socially meaningful pursuits, such as volunteering or parenting.
